# Restrictiveness matters

**DOI:** 10.3758/s13423-016-1194-3

**Published:** 2017-01-24

**Authors:** David Adger

**Affiliations:** Queen Mary University of London, London, UK

**Keywords:** Bayesian statistics, Knowledge

## Abstract

The Baysian Iterated Learning approach to language is consistent with generative grammar, but needs to be supplemented with specific cognitive constraints for empirical adequacy.

The Baysian Iterated Learning (BIL) research programme outlined by Kirby is clearly fertile, the model is elegant, and much of it is consistent with generative grammar. For decades now, theoretical linguists have been exploring what Chomsky (2005) calls “third factor effects” (a concept first raised as relevant to language 40 years before in Chomsky, 1965, p. 59). These are general laws of computational economy that play an important role in generative grammatical models. The BIL paradigm gives us a formal model of how such third-factor effects may shape linguistic diachronic change (cf. Niyogi & Berwick, 1997), though these must also interact with true sociocultural pressures (invasion, intermarriage, trade, slavery, reeducation, linguistic fashion and taboo, etc.; cf. Labov, 2000). What Kirby terms ‘cultural evolution’ is, actually, a noncultural part of language change: it is governed by computational law as opposed to the cultural forces identified in sociohistorical linguistics research.

While Kirby shows that BIL can computationally *model* the emergence of linguistic compositionality, that is not an argument that BIL *is* the etiology of compositionality. Indeed, the rapidity of creolization of Nicaraguan Sign Language mentioned at the end of Kirby’s paper, where third-generation signers already have highly complex compositional structures that are not in their input (Senghas 1995), argues against iterated learning, as there is simply not enough time for the relevant structure to emerge. Recent work shows that structures encoding agentivity and number do emerge in cases of transmission across generations in young sign languages (Horton, Goldin-Meadow, Coppola, Senghas, & Brentari, 2015), suggesting an important role for interaction in shaping particular aspects of grammars, but this happens so rapidly that specific cognitive structures seem to be involved in shaping the outcome. This conclusion is bolstered by the fact that core properties of language are extremely resilient, even in the total absence of relevant input (Coppola & Newport, 2005; Goldin-Meadow, 2003).

The BIL model builds in two constraints: compression of description (simplicity) and expressiveness. While important, these are not sufficient to capture crucial facts about human languages, whose systematicity is restricted in particular ways (a classic example is Chomsky’s question of why the syntactic rule of fronting cares about structure and not linear order; e.g. Chomsky, 1972, p. 61). Human language is full of phenomena where the most simple and expressive grammars are not the right ones. For example, Cinque (2013) shows that, over and over again, in different areas of grammar, the relationship between syntactic hierarchy and linear order has a logically possible but unattested slot. For example, while we find Demonstrative Numeral Adjective preceding the Noun we never find, in any language, the reverse order﻿ prenominally. The same effect holds elsewhere (adverbs, classes of adjectives, auxiliaries, etc.). The simplest and most expressive grammars should not have that missing slot. But the distribution of the data is elegantly captured by a grammatical model that maps scopal hierarchy monotonically to linear order, with a universal set of constraints on the relationship between scopal and syntactic position (e.g., Cinque, 2005; Abels & Neeleman, 2012). Culbertson and Adger (2014) argue that a historical explanation for such effects is implausible. That this effect appears across a range of grammatical structures, beyond nominals, argues for the necessity of a strong, rather than a weak, constraint on hierarchy to order mapping, incompatible with a BIL explanation.

The peculiarly restricted nature of grammatical structures across human languages, the rapidity of emergence of core properties of language in creolization, and the resilience of these properties in the absence of structure in the input, all suggest that the fact that human language, in general, is structured in restricted ways emerges from the brain at an individual level. The structures of particular languages, however, arise through interaction of language users within generations as well as through slow change across generations. This diachronic change involves third-factor effects of the sort captured by BIL, interacting with social and cultural pressures, but the change takes place within the constraints imposed by the nature of the human language capacity itself.
